# Retinol dehydrogenase 13 protects the mouse retina from acute light damage

**Published:** 2012-04-24

**Authors:** Haiyan Wang, Xiaofang Cui, Qing Gu, Yan Chen, Jia Zhou, Ying Kuang, Zhugang Wang, Xun Xu

**Affiliations:** 1 Department of Ophthalmology, Shanghai First People's Hospital, Affiliate of Shanghai Jiaotong University, Shanghai, PR China; 2 Department of Medical Genetics, E-Institutes of Shanghai Universities, Shanghai Jiaotong University School of Medicine, Shanghai, PR China; 3 Shanghai Research Center for Model Organisms, Shanghai, PR China

## Abstract

**Purpose:**

To investigate whether retinol dehydrogenase 13 (RDH13) can protect the retina from acute light-induced damage.

**Methods:**

We generated *Rdh13* knockout mice using molecular biologic methods and assessed the associated morphological and functional changes under room-light conditions by hematoxylin-eosin (H&E), transmission electron microscopy (TEM), and scotopic electroretinography. Then, the light-damage model was established by exposure to diffuse white light (3,000 lx) for 48 h. Twenty-four h after light exposure, H&E was used for the histological evaluation. The thickness of the outer-plus-inner-segment and the outer nuclear layer was measured on sections parallel to the vertical meridian of the eye. An electroretinography test was performed to assess the functional change. Furthermore, the impairment of mitochondria was detected by TEM. Finally, the expression of cytochrome c (CytC) and other apoptosis-related proteins was detected by western blot.

**Results:**

We found that there was no obvious difference in phenotype or function between *Rdh13* knockout and wild-type mice. In *Rdh13^−/−^* mice subjected to intense light exposure, the photoreceptor outer-plus-inner-segment and outer nuclear layer were dramatically shorter, and the amplitudes of a- and b-waves under scotopic conditions were significantly attenuated. Distinctly swollen mitochondria with disrupted cristae were observed in the photoreceptor inner segments of *Rdh13^−/−^* mice. Increased expression levels of CytC, CytC-responsive apoptosis proteinase activating factor-1 (Apaf-1) and caspases 3, and other mitochondria apoptosis-related genes (nuclear factor-kappa B P65 [*P65*] and B-cell lymphoma 2-associated X protein [*Bax*]) were observed in *Rdh13^−/−^* mice.

**Conclusions:**

*Rdh13* can protect the retina against acute light-induced retinopathy. The mechanism may involve inhibition of the mitochondrial apoptosis pathway.

## Introduction

**Figure 1:**
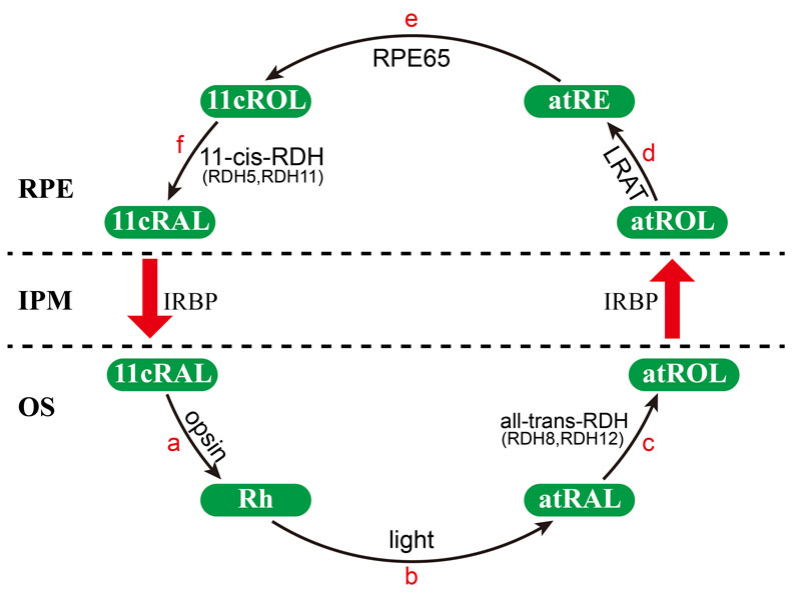
The visual cycle in the vertebrate retina. The classical visual cycle is a chain of biochemical reactions that are catalyzed by retinoid dehydrogenase/reductase (RDH) in photoreceptors or the retinal pigment epithelia (RPE) and are responsible for regenerating visual pigment following light exposure [28]. The visual process is initiated by the photoisomerization of 11-cis-retinal (11cRAL) to all-trans-retinal (atRAL). First, 11cRAL diffuses from the RPE to photoreceptor-rod outer segments (OS; rod outer segments, ROS; and cone outer segments, COS) and is coupled to opsin to generate rhodopsin (Rh; reaction a). Then, in the photoreceptor outer segments, the absorption of light by rhodopsin causes isomerization of the chromophore from the 11-cis form to the all-trans form (reaction b). The atRAL is reduced to all-trans-retinol (atROL) in the reaction catalyzed by an nicotinamide-adenine dinucleotide phosphate (NADPH)-dependent all-trans-retinal-specific dehydrogenase (all-trans-RDH, RDH8, RDH12; reaction c) [8,22]. Next, atROL diffuses to the RPE, where it is esterified to all-trans-retinyl-ester (atRE) in a reaction catalyzed by lecithin: retinol acyltransferase (LRAT; reaction d). The isomerization of atRE to 11cROL is catalyzed by RPE-specific 65 kDa protein (RPE65; reaction e), which is the key step in the retinoid visual cycle [29-31]. 11cROL is then oxidized by 11-cis-RDH (RDH5, RDH11) to 11cRAL to complete the retinoid cycle (reaction f). IPM, interphotoreceptor matrix; IRBP, inter-photoreceptor retinol binding protein.

Retinoid dehydrogenase/reductase (RDH) is a subfamily of the short-chain dehydrogenase/reductase family that participates in the metabolism of steroids, prostaglandins, and retinoids [1,2] and performs critical oxidation-reduction reactions during the retinoid cycle (Figure 1). RDH11–RDH14 share sequence similarity and RDH11 is strongly expressed in the retinal pigment epithelia (RPE) [3,4]; the other three all localize to the photoreceptor inner segment [5,6]. It has been demonstrated that 11-cis-RDHs (RDH5, RDH11) can catalyze the oxidation of 11-*cis-*retinol to 11-*cis-*retinal [7]. All-trans-RDHs (RDH8, RDH12, and RDH14) have very similar properties and can catalyze the reduction of all-trans-retinal to all-trans-retinol [5,8-10]. Haeseleer reported that RDH13 lacks RDH activity [5]. Recently, Belyaeva [11] showed that purified RDH13 can recognize all-trans-retinaldehydes as substrate in vitro, with nicotinamide-adenine dinucleotide phosphate (NADPH) as the preferred cofactor, and can exhibit catalytic activity as a reductase. Kinetic analysis revealed that RDH13 exhibited substrate and cofactor specificity similar to that of RDH11, RDH12, and RDH14, but exhibited greater catalytic efficiency in the reduction of all-trans-retinal to all-trans-retinol than in the oxidation of these compounds [11].Thus, RDH13 is considered to participate in the retinoid cycle, and it may be involved in the clearance of all-trans-retinal.

This clearance function of RDH13 is very important. During the visual cycle, the buildup of some photoisomerized chromophores can be toxic. Such is the case when all-trans-retinal is produced but cannot be cleared efficiently from the photoreceptors. This occurs because, in contrast to retinol, which has been found to be protective against oxidative damage [12], all-trans-retinal is detrimental. The delayed clearance of all-trans-retinal leads to the accumulation of its condensation products, diretinoid-pyridinium-ethanolamine (A2E) and all-trans-retinal dimer (RALdi), which are both associated with progressive retinal degeneration [13-15]. In this way, RDH13’s clearance of all-trans-retinal is speculated to protect the retina from all-trans-retinal-mediated retinal degeneration, especially acute light-induced retinopathy, which is mainly related to the delayed clearance of all-trans-retinal [16]. Mice lacking proteins critical for all-trans-retinal clearance from photoreceptors can develop severe RPE/photoreceptor dystrophy [17]. Here, we disrupted the *Rdh13* gene in mice to investigate whether there were associated morphological and functional changes to the retina and to determine whether *Rdh13* could protect the retina from intense light damage using biochemical and electrophysiological measurements.

## Methods

All of the procedures involving animals adhered to the Association for Research in Vision and Ophthalmology statement for the use of animals in ophthalmic and vision research.

### Rdh13 knockout mouse generation and maintenance

**Figure 2:**
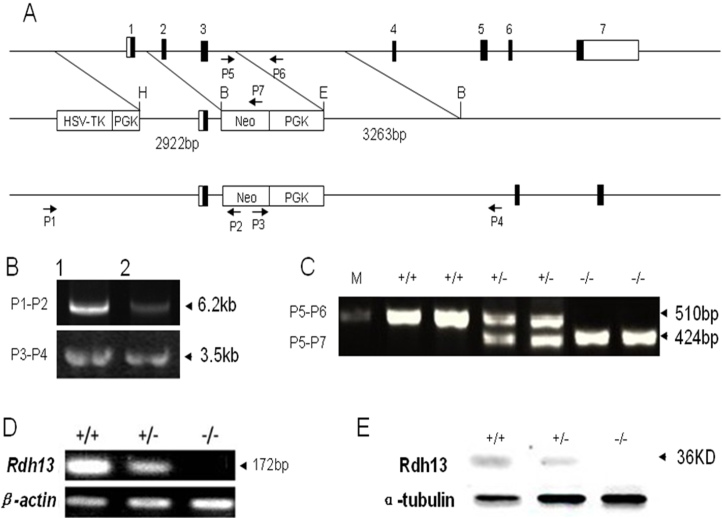
Generation of *Rdh13* knockout mice. **A**: This is the graphic representation of the *Rdh13* gene knockout strategy for the deletion of *Rdh13* exons 2 and 3 in embryonic stem cells. Exons are shown in boxes. The targeting vector was designed to delete exon 2 and exon 3. The targeting vector contained a 2.9 kb 5′ arm and 3.2 kb 3′ arm. PGK-Neo and HSV-TK cassettes were used for positive and negative selections, respectively. The genomic positive of the PCR primers for genotyping are indicated by arrows. **B**: Genomic DNA from ES cell clones was isolated and analyzed by PCR. The successfully targeted embryonic stem cell DNA was amplified into 6.2 kb and 3.5 kb products for the 5′ arm and 3′ arm, respectively. **C:** The genotype of *Rdh13^+/+^*, *Rdh13^+/−^*, and *Rdh13^−/−^* mice was detected by PCR. **D**: *Rdh13* transcripts in mouse liver from *Rdh13^+/+^*, *Rdh13^+/−^*, and *Rdh13^−/−^* mice was analyzed by reverse-transcription PCR. **E**: The expression pattern of RDH13 protein in *Rdh13^+/+^*, *Rdh13^+/−^*, and *Rdh13^−/−^* mouse liver was revealed by western blot.

A targeting vector was constructed by replacing the mouse *Rdh13* genomic 1,661 bp fragment, covering exons 2 and 3, with the 1,904 bp phosphoglycerate kinase-neomycin resistance cassette (PGK-Neo cassette) for positive selection. An external herpes simplex virus-1-thymidine kinase cassette (HSV-TK cassette) was used for negative selection (Figure 2A). The targeting vector contained 2.9 kb of homologous DNA upstream to the PGK-Neo cassette and 3.2 kb downstream as homologous recombination arms. The embryonic stem (ES) cells that had undergone homologous recombination were identified by PCR (Figure 2B) using two pairs of primers, whose direction and position is depicted in Figure 2A. The primers used for the 5′-arm recombination were (P1) 5′-CTT CTG CTT CTT GCC TAG TTC TTC TCA-3′ and (P2) 5′-AAT TGC ATC GCA TTG TCT GAG TAG G-3′. The 3′-arm primers were (P3) 5′-CCA GAG GCC ACT TGT GTA GCG-3′ and (P4) 5′-GAA GCA AAG AAC CAA CCC CTC TGA-3′. The correctly recombined ES cells were subsequently microinjected into blastocysts, which were in turn implanted into pseudopregnant female recipients to generate chimeric mice. The F1 mice with germ-line transmission of the *Rdh13* knockout (KO) allele were heterozygous. These heterozygous mice were identified by PCR using mouse tails and primers depicted in Figure 2A, (P5) 5′-CAG GAG GCA ACG TCA TTC TG-3′, (P6) 5′-GCT CAA TGA CAC TCC AGC AA-3′, (P7) 5′-TGG CTG GAC GTA AAC TCC TC-3′.

The mutant mice were maintained on a mixed 129Sv/C57BL/6 background during the earlier generations. Then the mice were backcrossed with wild-type (WT) line 129SV more than ten generations to ready them for experimentation. Mice were housed under specific pathogen-free (SPF) conditions at a constant room temperature of 22–24 °C with a 12 h:12 h light-dark cycle. The animals were provided with ad libitum access to food and water.

Total RNA was extracted using TRIzol (Invitrogen Life Technologies, Carlsbad, CA) reagent according to the manufacturer’s instructions. First-strand cDNA was synthesized from 1 µg of total RNA with random 6-mer primers at 25 °C for 10 min and 42 °C for 1 h. One microliter of the reverse transcription was used as the PCR template.

### Immunofluorescence and western blot

#### Immunofluorescence

Retinal samples were fixed with 4% paraformaldehyde in phosphate-buffered saline and processed for paraffin embedding. Sections were deparaffinized, rehydrated, and then treated with 0.3% hydrogen peroxide in methyl alcohol for 20 min to block endogenous peroxidase activity. After antigen retrieval in citrate buffer by autoclave boiling for 15 min, the sections were blocked for 1 h with 2% normal goat serum and incubated with anti-RDH13 serum at 4 °C overnight. The next day, the secondary rhodamine conjugated antibody (at 1:1,000 dilution) was applied for 2 h at room temperature. Slides were mounted and visualized under a confocal laser scanning microscope (Leica, Heidelberg, Germany).

#### Western blot

For western blots, retinas were isolated and homogenized in radioimmunoprecipitation assay (RIPA) buffer (Beyotime Biotech, Nantong, Jiangsu, China). Protein concentration was determined with the BCA protein assay (Beyotime Biotech), and the supernatants were then separated by 12% sodium dodecyl sulfate PAGE (SDS–PAGE) and transferred to polyvinylidene fluoride (PVDF) membranes (Millipore, Watham, MA). The membrane was blocked in Tris-buffered saline with 0.05% Tween-20 and 5% nonfat milk and then rinsed and incubated with primary antibody at 4 °C overnight. After incubation with a horseradish-peroxidase-conjugated anti-rabbit immunoglobulin G (IgG; Cell Signaling Technology, Danvers, MA) or anti-mouse IgG (Chemicon, Temecula, CA) for 2 h at room temperature, the membranes were evaluated using enhanced chemiluminescence according to the manufacturer’s instructions. The following antibodies were used for western blotting: 1:4,000 RDH13 anti-serum, rabbit polyclonal anti-Fas (Santa Cruz Biotech, Santa Cruz, CA), mouse monoclonal anti-Bax (Sigma Chemical, St. Louis, MO), mouse monoclonal anti-caspase 3 (Santa Cruz Biotech), recombinant rabbit monoclonal anti-TNF-α (Molecular Probes, Eugene, OR), mouse monoclonal anti-P65 (Molecular Probes), mouse monoclonal anti-Apaf-1 (Santa Cruz Biotech), mouse monoclonal anti-cytochrome c (BD PharMingen, San Diego, CA), and mouse monoclonal anti-cytochrome oxidase IV (Molecular Probes).

### Histology and transmission electron microscopy

#### H&E

For histology, the eyecups were fixed in 4% paraformaldehyde for 20 h, incubated in 20% sucrose, and then embedded in paraffin. Subsequently, fixed eyes were sectioned at a thickness of 5 µm and then stained with hematoxylin and eosin solution (H&E; Sigma). An Axioplan 2 imaging microscope (Zeiss, Jena, Germany) was used for histological evaluation of the sections, and color micrographs were obtained at 5× and 400× magnification using Zeiss Axiovision software (Zeiss). The thickness of the outer-plus-inner-segment (from the external limiting membrane to the pigment epithelium) and the outer nuclear layer was measured on sections parallel to the vertical meridian of the eye at a distance of 1,400 µm from the optic nerve by the Zeiss Axiovision software and plotted with standard deviations (SDs).

#### Transmission electron microscopy

For transmission electron microscopy (TEM), the mice eyecup sections were fixed in 2.5% glutaraldehyde in 1.0 M piperazine-1,4-bisethanesulfonic acid (PIPES), pH 7.4, at 4 °C for 4 h and then washed three times with 0.1 M sodium phosphate, pH 7.3 and post-fixed with 1% osmium tetroxide in 0.1 M sodium phosphate, pH 7.3, for 3 h at room temperature. The eyecups were dehydrated using an ethanol and acetone series and embedded with pure epoxy embedding medium. Then, the eyecups were sectioned at 70 nm with an LKB-5 microtome (LKB, Stockholm, Sweden) and stained with 3% uranium acetate and lead citrate before viewing with a JEM-1230 transmission electron microscope (JEOL, Tokyo, Japan).

### Electroretinography

Ganzfeld electroretinography (ERG) was used to evaluate *Rdh13^+/+^* and *Rdh13^−/−^* mice (Tomey EP-1000 Erlangen, Germany). After overnight dark adaptation, the mice were anesthetized by intraperitoneal injection using 1% pentobarbital sodium (70 mg/kg; Sigma Chemical) under dim red light. The pupils were dilated with 0.5% Mydrin-P (Tropicamide/Phenylephrine; Santen Pharmaceutical, Japan). A contact-lens electrode was placed on the center of the cornea with a drop of sodium hyaluronate, and a reference electrode and ground electrode were placed in the middle of the lower eyelid and near the tail, respectively. The light intensity (2.5 cd/m^2^) was calibrated and computer controlled, and the preamplifier bandwidth was set at 0.3–300 Hz. The mice were placed in a Ganzfeld chamber, and the scotopic response to flash stimuli was measured in both eyes simultaneously.

### Light damage

Light damage was induced as described in previously published work [18,19] with slight modifications. Before light exposure, the mice were dark-adapted for 48 h, and the pupils were dilated under dim red light with 0.5% Mydrin-P. Light damage to *Rdh13^+/+^*and *Rdh13^−/−^* mice was induced by exposure to diffuse white fluorescent light (TLD-30W/965 tubes; Philips, Hamburg, Germany) for 48 h (lights on at 11:00 AM) with an intensity of 3,000 lx. After being exposed to bright light, the mice were maintained in dark-adaptation conditions for 24 h. Analyses of morphology, function, and apoptosis were then performed (n=18, each group). *Rdh13^+/+^*and *Rdh13^−/−^* mice without light exposure were assigned to the control group.

### Terminal deoxynucleotidyl transferase dUTP nick-end labeling

Paraffin-embedded retinal sections were deparaffinized, rehydrated, and pretreated with Proteinase K (Roche, Mannheim, Germany). Terminal deoxynucleotidyl transferase dUTP nick-end labeling (TUNEL) assays were performed with an In Situ Cell Death Detection Kit, TMR red (Roche), according to the manufacturers’ protocols. Slides were mounted in Vectashield fluorescence mounting medium (Vector Laboratories, Burlingame, CA) with DAPI (Sigma Chemical) for nuclear staining and were visualized under a confocal laser scanning microscope (Leica).

### Statistical analysis

The results were plotted as the mean±SD. Differences were assessed by the two-tailed Student's *t*-test using SPSS 16.0 software (SPSS; Chicago, IL); p<0.05 was considered statistically significant.

## Results

### Disruption of *Rdh13*

The gene-targeting strategy employed resulted in the deletion of *Rdh13* exons 2 and 3 (Figure 2A). Genomic DNA from ES cell clones was isolated and analyzed by PCR, and the successfully targeted ES cell DNA was amplified to 6.2 kb and 3.5 kb products for the 5′ arm and the 3′ arm, respectively (Figure 2B). Genotypes were determined by PCR analysis, which showed that the sizes of the WT and KO allele products were 510 bp and 424 bp, respectively (Figure 2C). No *Rdh13* transcripts were detected in the livers of *Rdh13* KO mice by reverse-transcription (RT)-PCR analysis (Figure 2D). Western-blot analysis of RDH13 levels in the livers of *Rdh13^+/+^*, *Rdh13^+/−^*, and *Rdh13^−/−^* mice showed that protein levels were reduced in heterozygous animals and absent in *Rdh13* KO animals (Figure 2E). After the ablation of *Rdh13*, we examined the basic phenotype of *Rdh13* KO mice and found no abnormalities in genotype distribution, sex ratio, bodyweight, or body length compared with their WT littermates. Furthermore, no significant impairment in reproduction capability was observed.

### Characterization and histology of the Rdh13 knockout mice

**Figure 3:**
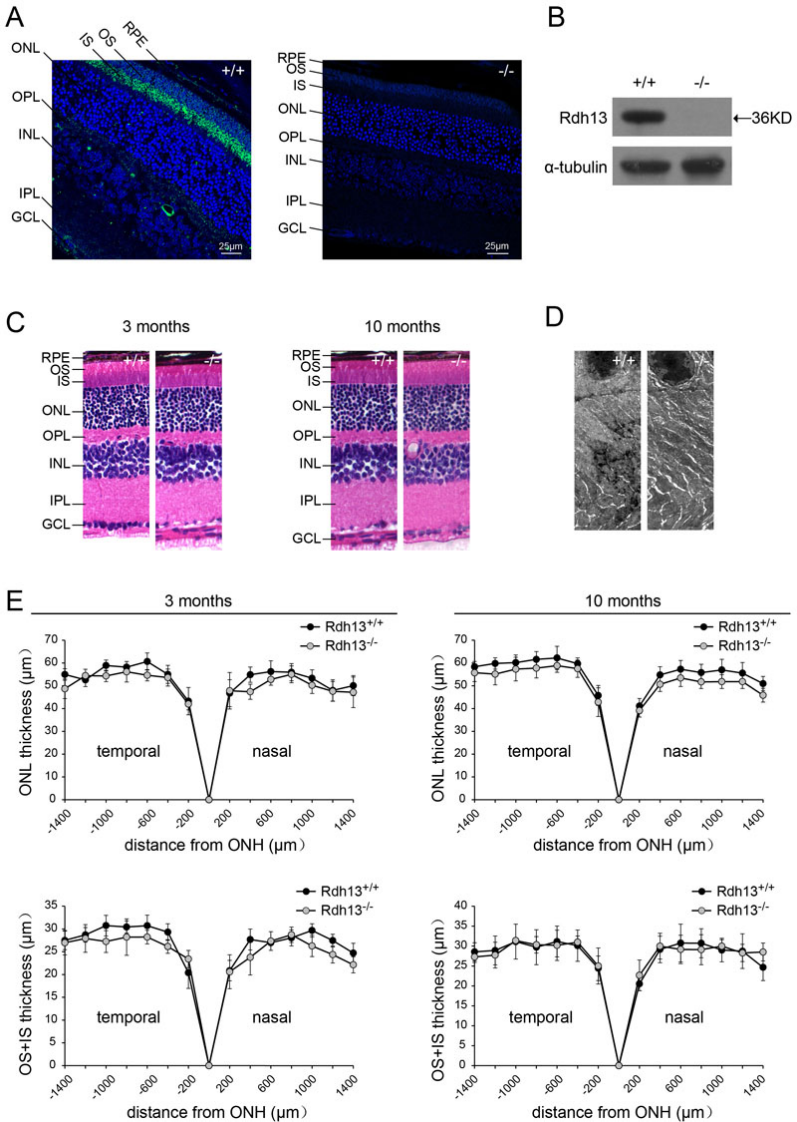
Localization, retinal histology, and thickness measurements for the *Rdh13* knockout mice. **A**: Immunofluorescence localization of *Rdh13* (green) was shown in 3-month-old *Rdh13^+/+^* and *Rdh13^−/−^* retina paraffin sections. *B*: western blot analysis of RDH13 protein in wild-type (WT) and homozygous mice revealed that there was no expression of *Rdh13* in the retinas of *Rdh13* knockout mice. **C**: Semi-thin sections of WT and homozygous mice retinas revealed no major differences in retinal histology at 3 and 10 months of age. **D**: Transmission electron microscopy (TEM) of the photoreceptor outer and inner segments and the outer nuclear layer in *Rdh13^+/+^* and *Rdh13^−/−^* mice at 10 months of age revealed no apparent abnormalities. **E**: The outer-plus-inner-segment and outer nuclear layer thickness for *Rdh13^−/−^* and WT mice at the ages of 3 months and 10 months was valued. Values were mean±SD (n=5, each group). There were no statistically significant differences between the two genotypes at any distance point. ONH, optic nerve head; RPE, retinal pigment epithelia; OS, outer segments; IS, inner segments; ONL, outer nuclear layer; OPL, outer plexiform layer; INL, inner nuclear layer; IPL, inner plexiform layer; GCL, ganglion cell layer.

For *Rdh13* localization in mouse eyes, immunofluorescence analysis showed that *Rdh13* was expressed in the photoreceptor inner segment layer, with no apparent expression in the RPE, photoreceptor outer segments, or outer nuclear layer in WT mice (Figure 3A). *Rdh13* expression was abolished in the retinas of *Rdh13* KO mice, as determined by immunofluorescence (Figure 3A) and western blot (Figure 3B). Light micrographs of retina sections from *Rdh13* KO and WT mice revealed no major differences in retinal histology at 3 and 10 months of age (Figure 3C, at 3 months and 10 months), with normal lamination and numbers of cells. Similarly, TEM at 10 months of age revealed no apparent abnormalities in retinal architecture (Figure 3D). There was no evidence of any abnormality in Bruch’s membrane, increased pigmented body and lipofuscin accumulation in retinal pigment epithelium cells, or photoreceptor debris. The thickness of the outer-plus-inner-segment and outer nuclear layer of the retina was similar at 1,400 µm to that of the optic nerve head for both genotypes at 3 months and 10 months of age (Figure 3E).

### ERG and apoptosis of Rdh13^−/−^ mice

**Figure 4:**
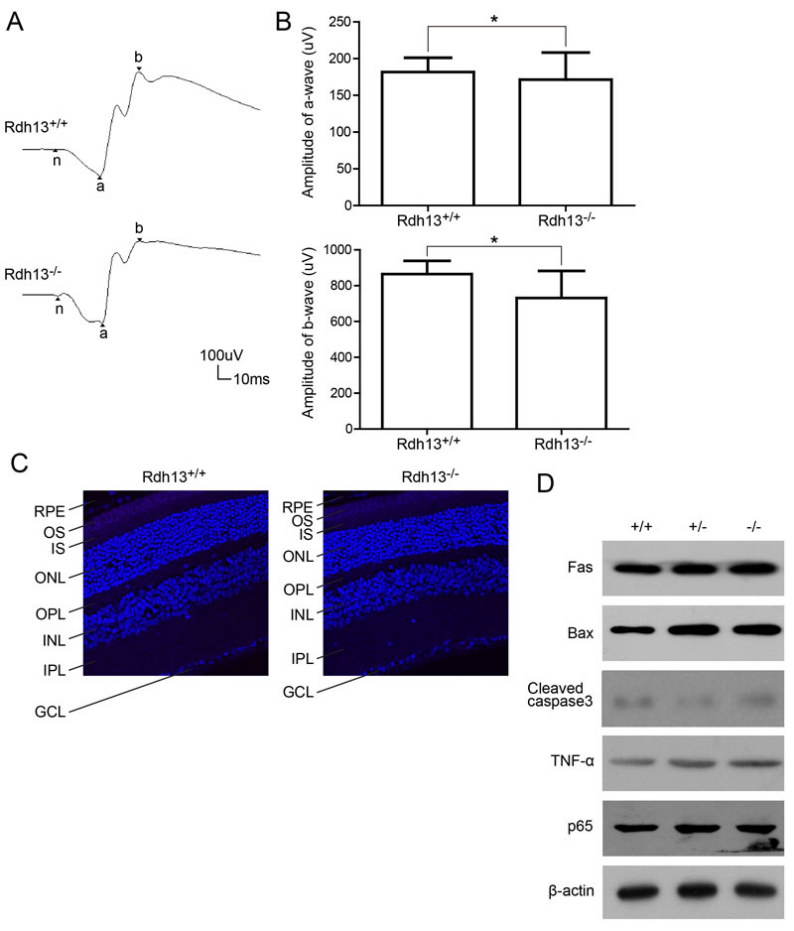
Full-field electroretinogram responses and apoptosis detection in *Rdh13^+/+^* and *Rdh13^−/−^* mice. **A**: The scotopic electroretinogram responses of *Rdh13^+/+^* and *Rdh13^−/−^* mice at 10 months of age were recorded. **B**: The amplitudes of a- and b-waves for either genotype was plotted as the mean±SD (n=5, each group), *: p>0.05. **C**: The terminal deoxynucleotidyl transferase dUTP nick-end labeling (TUNEL) staining showed that there was no obvious apoptosis in both *Rdh13^+/+^* and *Rdh13^−/−^* mice at 10 months of age. **D**: Apoptosis genes expression in *Rdh13^+/+^*, *Rdh13^+/−^*, and *Rdh13^−/−^* mice was detected by western blots. There was no increased expression level of apoptosis genes in all genotypes, which was in accordance with the result of TUNEL.TNF-α, tumor necrosis factor alpha; Fas, TNF receptor superfamily member 6; Bax, B-cell lymphoma 2-associated X protein; P65, nuclear factor-kappa B P65; RPE, retinal pigment epithelia; OS, outer segments; IS, inner segments; ONL, outer nuclear layer; OPL, outer plexiform layer; INL, inner nuclear layer; IPL, inner plexiform layer; GCL, ganglion cell layer.

To evaluate whether visual function was impaired in *Rdh13* KO mice, Ganzfield ERG was performed on 10-month-old *Rdh13^+/+^* and *Rdh13 ^−/−^* mice. The typical ERG records are displayed in Figure 4A. There was no significant difference in the amplitudes of the a- and b-waves of the scotopic ERG response between dark-adapted *Rdh13^+/+^* and *Rdh13^−/−^* mice (a-wave amplitude: 181.75±19.44 versus 171.32±36.95 μv, p>0.05; b-wave amplitude: 864.45±74.18 versus 730.95±151.52 μv, p>0.05; Figure 4B). Thus, *Rdh13* deletion did not have a significant effect on the rod-mediated light response. TUNEL detection showed that there was no obvious apoptosis in 10-month-old *Rdh13^−/−^* mice (Figure 4C), as determined by western blot, which showed no increased expression levels of caspases 3, P65, or other mitochondria apoptosis-related genes (Fas, TNF-α and Bax; Figure 4D).

### Light damage in *Rdh13*^-^/^-^ mice exposed to intense light

**Figure 5:**
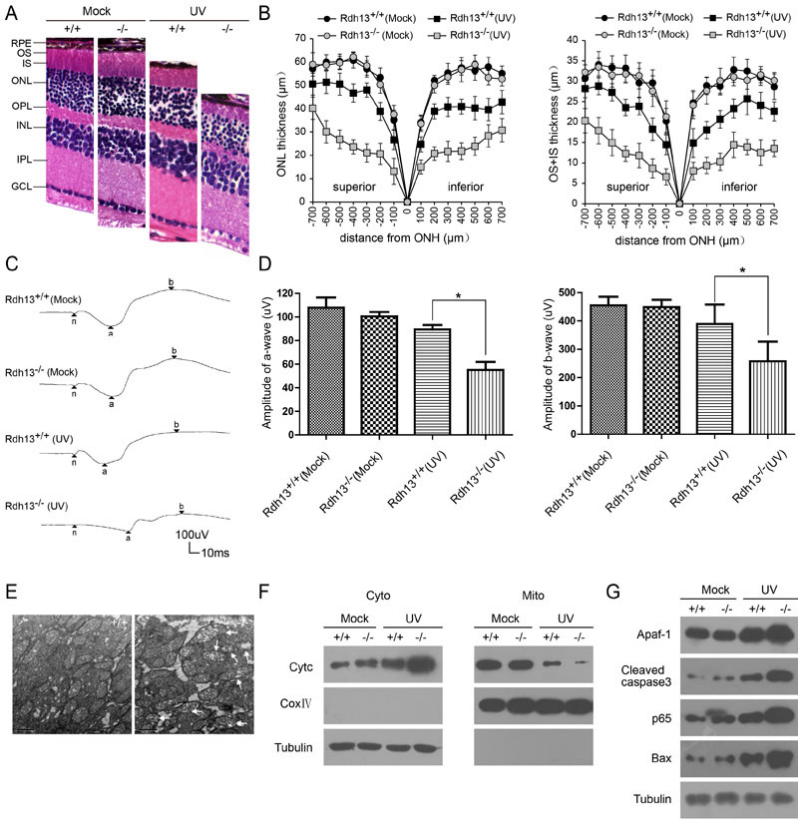
Light damage in *Rdh13^-^*^/-^ mice. Light damage was induced in *Rdh13^+/+^* and *Rdh13^−/−^* mice by 48 h exposure to diffuse white light (3,000 lx). Twenty-four h dark-adaption after light exposure, and morphological and functional assays were performed as described in Methods. **A**: Hematoxylin-eosin (H&E) staining showed that the outer-plus-inner-segment and outer nuclear layers of the retina from *Rdh13^−/−^* mice that were exposed to light obviously disintegrated. **B**: The thicknesses of the outer-plus-inner-segment and outer nuclear layers of all genotypes exposed to the light and the control group was valued. Values were mean±SD (n=5, each group). There were statistically significant differences in the thickness of the outer-plus-inner-segment and outer nuclear layer between the light exposed *Rdh13^−/−^* mice and the other three groups at any distance point. **C**: The scotopic electroretinogram response of *Rdh13*^+/+^ and *Rdh13^−/−^* mice, which were recorded in all groups. **D**: The amplitudes of a- and b-waves in all genotypes was plotted as the mean±SD (n=5, each goup); *: p<0.05. **E**: Mitochondria in photoreceptor inner segments of *Rdh13^+/+^* and *Rdh13^−/−^* mice exposed to the light were detected by transmission electron microscopy. Distinctly swollen mitochondria with disrupted cristae were observed in *Rdh13^−/−^* mice (arrows). **F** and **G**: Cytochrome c (CytC) and apoptotic gene expression in all groups were analyzed by Western Blot, which revealed that levels of CytC, apoptosis proteinase activating factor-1 (Apaf-1), cleaved caspase 3, nuclear factor-kappa B P65 (p65) and B-cell lymphoma 2-associated X protein (Bax) were clearly increased in the cytoplasm of *Rdh13^−/−^* mice; ONH, optic nerve head; RPE, retinal pigment epithelia; OS, outer segment; IS, inner segment; ONL, outer nuclear layer; OPL, outer plexiform layer; INL, inner nuclear layer; IPL, inner plexiform layer; GCL, ganglion cell layer; UV, ultraviolet.

When *Rdh13^−/−^* mice were exposed to 3,000 lx of white light for 48 h, the outer-plus-inner-segment and outer nuclear layers around the central area (500 μm from the optic nerve head), where the light of strongest intensity penetrates, disintegrated (Figure 5A). The thickness values of the outer-plus-inner-segment were reduced compared to those observed in the *Rdh13^+/+^*mice, especially the outer segment (100 μm superior: 6.57±1.02 versus 14.43±1.45 μm, p<0.05; 100 μm inferior: 7.64±1.36 versus 14.79±2.64 μm, p<0.05; 500 μm superior: 14.95±2.41 versus 27.27±1.14 μm, p<0.05; 500 μm inferior: 13.88±1.11 versus 25.70±3.16 μm, p<0.05) (Figure 5B). There was also a distinct difference in the thickness of the outer nuclear layer between the *Rdh13^−/−^* and *Rdh13^+/+^*mice (100 μm superior: 13.07±4.21 versus 26.62±2.84 μm, p<0.05; 100 μm inferior: 15.01±1.39 versus 24.68±1.70 μm, p<0.05; 500 μm superior: 26.84±2.78 versus 50.52±3.63 μm, p<0.05; 500 μm inferior: 23.86±2.47 versus 40.25±3.71 μm, p<0.05; Figure 5B). These findings were matched to data obtained by the full-field ERG, which revealed markedly decreased mean amplitudes of a- and b-waves under scotopic conditions in the *Rdh13^−/−^* mice compared with the *Rdh13^+/+^*mice (a-wave amplitude: 55.08±6.73 versus 89.38±3.86 μv, p<0.05; b-wave amplitude: 257.73±69.57 versus 389.30±68.25 μv, p<0.05; Figure 5C,D). TEM showed that the mitochondria in the photoreceptor inner segments of the *Rdh13^−/−^* mice were distinctly swollen and contained disrupted cristae. In contrast, the morphology of the mitochondria in the *Rdh13^+/+^* mice was only minimally affected (Figure 5E). Western blot analysis revealed that levels of cytochrome C (CytC), apoptosis proteinase activating factor-1 (Apaf-1), caspase 3, p65, and Bax were clearly increased in the cytoplasm of *Rdh13^−/−^* mice (Figure 5F,G).

## Discussion

During the visual cycle, there is one important catalytic step involving the enzymatic reduction of all-trans-retinal to all-trans-retinol, and it is performed in photoreceptors by all-trans-RDH [20]. This process is not only essential for the retinoid cycle but can also protect the retina against oxidative damage caused by the accumulation of all-trans-retinal, which is also of great significance in the occurrence of retinal degeneration [17]. Among the all-trans-RDH family members participating in the reduction of all-trans-retinal, RDH8 and RDH12 have been shown to be responsible for clearing all-trans-retinal from the outer and inner segments of photoreceptor cells, respectively [21]. Mutations in RDH8 and RDH12, either singly or in combination, can cause severe retinal dystrophy [17,22,23]. Recent observations suggest that RDH13 also participates in the retinoid cycle and catalyzes the reduction of all-trans-retinal to all-trans-retinol [11]. The role of RDH13 is not clear, and whether *Rdh13* is associated with severe retinal degeneration remains to be clarified. In our investigation of *Rdh13*-KO and WT mice maintained under room-light conditions, no obvious pathological or abnormal electrophysiological response was observed in *Rdh13^−/−^* mice, which meant that loss of RDH13 failed to lead to progressive retinal degeneration. This was probably because the deficiency of RDH13 was compensated for by other dehydrogenases and reductases, such as RDH8 and RDH12.

Next, we studied whether *Rdh13* plays a role in the abnormal increase in levels of all-trans-retinal. The light-damage model, which is mainly related to the accumulation of all-trans-retinal, has been established. The appropriate morphological and functional assays have been performed. Retinal visual function and morphology were preserved in *Rdh13^+/+^* mice but were dramatically disturbed in *Rdh13^−/−^* mice, which may be because the photoreceptor outer-plus-inner-segment and outer nuclear layers were shorter. Thus, *Rdh13* seemed to reinforce the anti-acute light damage vulnerability of the retina and inhibit retinal degeneration caused by intense light exposure.

Further observation showed that, 24 h after light exposure, mitochondria in the photoreceptor inner segment were distinctly swollen and contained disrupted cristae in the *Rdh13^−/−^* mice (Figure 5E). This impairment clearly preceded the major burst of photoreceptor apoptosis (Figure 5A). The western blot analysis revealed that levels of CytC increased markedly in the cytosol of the *Rdh13^−/−^* mice (Figure 5F). The release of CytC from mitochondria to the cytosol contributes to changes in the mitochondrial membrane and triggers the common apoptotic pathway, which involves a cascade of caspases [24-26] (Figure 5G). The rate of mitochondrial apoptosis was consistently lower in the retinas of *Rdh13^+/+^* mice. Thus, it appears likely that *Rdh13* levels were sufficient to inhibit mitochondrial apoptosis and to protect the retina against mitochondria-associated cell death. RDH13 has been found to be a mitochondrial protein rather than a microsomal protein [27], and the submitochondrial localization of RDH13 is on the outer side of the inner mitochondrial membrane [11]. One may assume that the localization of RDH13 at the entrance to the mitochondrial matrix may serve as a barrier protecting the mitochondria against light-induced oxidative damage, and *Rdh13* may interact with certain mitochondrial membrane proteins to exert its protective effects. Further studies are necessary to explore this potential function of *Rdh13* in mitochondria.

In conclusion, the evidence suggests that *Rdh13* protects specifically against light-induced apoptosis in photoreceptors. The protective properties of *Rdh13* are associated with the inhibition of mitochondria-associated cell death.
